# Impact of COVID-19 lockdown on maternal psychological status, the couple’s relationship and mother-child interaction: a prospective study

**DOI:** 10.1186/s12884-022-05063-6

**Published:** 2022-09-26

**Authors:** Sylvie Viaux-Savelon, Paul Maurice, Alexandra Rousseau, Chloe Leclere, Manon Renout, Laura Berlingo, David Cohen, Jean-Marie Jouannic

**Affiliations:** 1 Department of Child and Adolescent Psychiatry, AP-HP.Sorbonne Université, Pitié-Salpêtrière Hospital, Paris, France; 2 Fetal Medicine Department, AP-HP.Sorbonne Université, Armand Trousseau Hospital, Paris, France; 3 AP-HP.Sorbonne Université, URCEST, Paris, France; 4 AP-HP.Sorbonne, Obstetrics and Gynecology Department, Tenon Hospital, Paris, France; 5 AP-HP.Sorbonne, Obstetrics and Gynecology Department, Pitié-Salpêtrière Hospital, Paris, France

**Keywords:** Covid-19, Pregnancy, Lockdown, Depression, Anxiety, Post-traumatic stress, Mother-child interaction

## Abstract

**Background:**

To compare the rate of postpartum depression (PPD) during the first COVID-19 lockdown with the rate observed prior to the pandemic, and to examine factors associated with PPD.

**Methods:**

This was a prospective study. Women who gave birth during the first COVID-19 lockdown (spring 2020) were offered call-interviews at 10 days and 6–8 weeks postpartum to assess PPD using the Edinburgh Postnatal Depression Scale (EPDS). Post-traumatic symptoms (Perinatal Post-traumatic Stress Disorder Questionnaire, PPQ), couple adjustment, and interaction and mother-to-infant bonding were also evaluated. The observed PPD rate was compared to the one reported before the pandemic. Factors associated with an increased risk of PPD were studied. The main outcome measures were comparison of the observed PPD rate (EPDS score > 12) to pre-pandemic rate.

**Results:**

Of the 164 women included, 27 (16.5% [95%CI: 11.14–23.04]) presented an EPDS score > 12 either at 10 days or 6–8 weeks postpartum. This rate was similar to the one of 15% reported prior to the pandemic (*p* = 0.6). Combined EPDS> 12 or PPQ > 6 scores were observed in 20.7% of the mothers [95%CI: 14.8–0.28]. Maternal hypertension/preeclampsia (*p* = 0.007), emergency cesarean section (*p* = 0.03), and neonatal complications (*p* = 0.008) were significantly associated with an EPDS> 12 both in univariate and multivariate analysis (OR = 10 [95%CI: 1.5–68.7], OR = 4.09[95%CI: 1.2–14], OR = 4.02[95%CI: 1.4–11.6], respectively).

**Conclusions:**

The rate of major PPD in our population did not increase during the first lockdown period. However, 20.7% of the women presented with post-traumatic/depressive symptoms.

**Trial registration:**

NCT04366817.

## Background

The COVID-19 epidemic has had a major impact on societal organization and on the organization of healthcare systems. Both maternal and fetal outcomes have worsened during the pandemic and great disparities have been highlighted from country to country according to the level of resources.^1^ Few studies have evaluated the impact of this crisis on the psychological well-being of women during the perinatal period. While an overall increase in anxiety in the prenatal and postnatal periods has been reported, studies of changes in postpartum depression (PPD) rates are scarce and have yielded conflicting results [1–4].

PPD varies greatly from one geographical area to another [5, 6]. In France, the prevalence of PPD varies between 10 and 20% [7] and was close to 15% prior to the pandemic in the Parisian population [8]. The psychological well-being of pregnant women can influence their subjective experience of childbirth [9], their relationship with their partner [10], and mother-child bonding. Maternal stress during pregnancy may also cause emotional-neuro developmental disorders in the offspring [11–13].

During the first wave of the COVID-19 epidemic, most affected countries decided to restrict or even ban visits to hospitalized patients. In France, most maternity wards decided to allow the presence solely of the pregnant woman’s partner in the delivery room and to prohibit visits during the postnatal hospital stay [14]. The separation of women from their relatives during this period exposed them to a greater psychological vulnerability [6, 15]. Moreover, when women returned home, the implemented lockdown measures prevented visits by family members and limited face-to-face management by caregivers.

In anticipation of a situation of greater vulnerability related to the health crisis, the three maternity units of Sorbonne University, Paris, France, set up an organization to support women who gave birth during the first lockdown [14]. All women who gave birth in these units were offered a follow-up through telephone interviews with psychologists after they returned home. The interviews were conducted at Day 10 and 6–8 weeks postpartum, with the goal to identify patients at risk of developing PPD, and to organize, when necessary, flexible care plans for these mothers.

The main aim of our study was to compare the rate of PPD during the period of the first 2020 lockdown with the rate observed prior to the COVID-19 pandemic, and to examine factors associated with PPD. The secondary objectives were to study the impact on the couples’ relationships, and the mother-child interaction.

## Methods

### Study design and participants

We conducted a prospective study involving women delivering a single live birth in one of the three maternities of the Sorbonne University during the first strict lockdown in France from 27th March to 5th May 2020. The study was conducted in accordance with the Helsinki declaration and was approved by the local ethics committee (Institutional Review Board Ile de France II, approval 27,042,020 - ClinicalTrial.gov ID: NCT04366817). All participants gave written informed consent. The inclusion criteria were: maternal age ≥ 18 years, French social security registration, patients speaking and understanding French, and maternal post-delivery hospitalization in the conventional postnatal ward. The exclusion criteria were: women who did not speak French, mothers hospitalized in other units, and mothers of babies hospitalized in the Neonatal Intensive Care Unit.

Eligible participants were recruited by the midwives in the postnatal units and were offered the possibility of one telephone interview with a psychologist at Day 10 (±1 day) postpartum, and then another one 6 to 8 weeks later. Three attempts were made to call patients who failed to answer. The standardized interviews lasted approximately 45 minutes and were structured in two parts: (i) a structured interview with validated questionnaires, and (*ii*) a semi-structured interview about the mother’s experience of childbirth, the maternity ward, conditions of discharge, and the first days/weeks at home with the baby and their partner.

### Study measures

We extracted sociodemographic and obstetrical data from the obstetrical records. These included: (*i*) socioeconomic data (Marital/couple status, Educational Level, Profession, Current financial situation, Geographic origin), (*ii*) Obstetrical background: history of perinatal death, fetal malformation, intra-uterine growth retardation (IUGR), maternal-fetal infection, (*iii*) Maternal complications during the current pregnancy: hypertension, diabetes, hospitalization for threatened preterm labor, (*iv*) Maternal psychiatric background: history of PPD, history of mood or anxiety disorders, history of Post-Traumatic Stress Disorder (PTSD), and (*v*) Specific psychosocial contexts: precarity, conflict within the couple, single parent, transgenerational familial history of obstetrical pathologies.

The following validated self-questionnaires were used during the interviews to assess postpartum depressive and post-traumatic symptoms, couple satisfaction and mother-child interactions:

- 1). The Edinburgh Postnatal Depression Scale (EPDS) [7], which is a 10-item questionnaire specific to the postpartum period and results in a depression score. For this study, we used the score as a continuous variable. An EPDS score > 12 was used to define a major risk of PPD, and an EPDS between 10 and 12 a mild to major risk.
- 2). The Perinatal Posttraumatic Stress Disorder Questionnaire (PPQ) [16] (validated French version [17]), which evaluates post-traumatic stress reactions of parents undergoing a childbirth with a high perinatal risk. It consists of 14 items, which refer to the DSM-V criteria of PTSD. A threshold of 6 was used to define a high risk of perinatal post-traumatic syndrome [16].
- 3). The couple’s relationship (Dyadic Adjustment Scale; DAS-16) [18] is a self-questionnaire derived from the original 32-item DAS scale, which evaluates dyadic adjustment in the marriage [19]. The revised scale consists of 16 items relative to the quality of the couple’s relationship and includes two dimensions: the degree of accordance (DA), and the quality of interaction (QI). The participants are asked to indicate their responses in relation to their experiences in the preceding 2 weeks, on a 6-point Likert scale. The score is calculated as a summation of all the responses, and ranges from 16 to 96. Scores above 54 are considered to represent satisfactory adjustment. Some questions concern the frequency in which the couple laughs together, or the frequency with which the couple considers divorce. The last question asks, “In general, to what degree do you experience happiness in your relationship” on a scale from “extremely unhappy” to “extremely happy”.
- 4). The Mother-Infant Bonding Scale (MIBS) [20] was initially developed to detect disturbances of maternal feelings towards their newborns. It is a short, simple questionnaire, used after birth in the maternity ward by different medical professionals: midwives, obstetricians, pediatricians, nurses. The scores range from 0 to 24, with a higher score indicating an impaired mother-infant bond.

As part of this study, psychological or psychiatric support was offered to each woman with an EPDS score > 10 and/or a PPQ score > 6, or if the interview revealed clinical vulnerability indicators (e.g., expression of emotional distress, history of perinatal loss, separation from the partner) whatever the results of EPDS score.

### Statistical analysis

The study was designed to achieve 80% of power to detect at least a 5-point change in the proportion of major PPD compared to the expected proportion of 15% (15 vs. 20%), considering two-sided alpha risk of 5 and 5% of dropout. Therefore, 452 women needed to be included.

Continuous variables were described as mean and standard deviation or median and interquartile range, depending on the distribution of the variable. Qualitative variables were expressed as numbers and proportions.

The proportion of patients with major PPD during follow-up, defined by an EPDS score > 12, was calculated with its 95% confidence interval (exact method) and the exact binomial test was used to test the superiority of the proportion compared with the expected value of 15%.

The association between depression and baseline characteristics was studied using logistic regression. Unadjusted analysis was performed to select variables at the 20% threshold taking into account missing values and relationships between variables or redundancy.

Post hoc analysis also assessed the association between baseline characteristics and PPD and/or post-traumatic symptoms during follow-up (EPDS > 12 or PPQ > 6).

Spearman rank correlation was used to determine post hoc correlations between scores. Missing values were not replaced. All tests were two-sided and a *P*-value < 0.05 indicated statistical significance. Analyses were performed using SAS version 9.3, SAS institute Inc., Cary, USA.

## Results

### Patient flow and recruitment

**Fig. 1:**
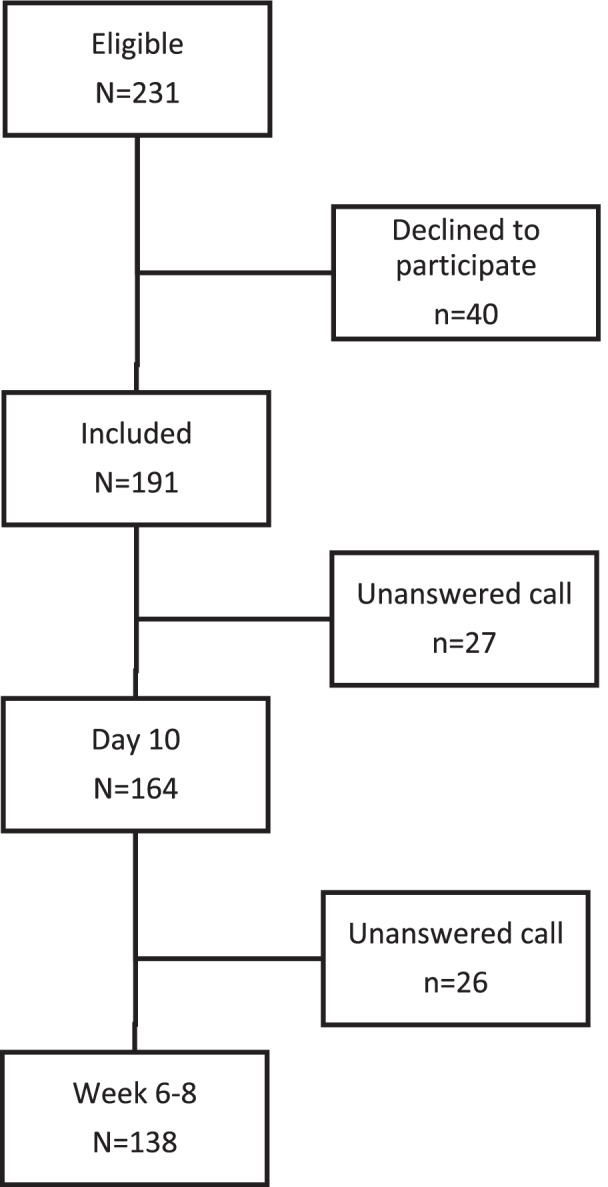
Flow chart of the study

Fig. 1 details the patient flow chart of the study. A total of 231 women were eligible for inclusion, and 191 agreed to participate. Of these, 164 had a call interview on Day 10 and 138 at 6–8 weeks postpartum.

### General characteristics of the population

The average maternal age was 34.1 years (range: 22–47 years) (Table 1). The education level of women was high. Most of the mothers (94.9%) were in a couple and overall there was a low level of previous psychiatric or obstetrical history. Social vulnerability was present for 6.1% of the mothers (migration 3.1%, accommodation in a hostel 3.7%). A context of conjugal conflict or violence was reported by 1.2% of the women. Finally, family isolation was reported by 26.7% of the mothers. Among the 15 (9.3%) women with psychiatric or social vulnerability, six (3.7%) had a history of psychiatric or neurologic pathology, three (1.8%) of perinatal depression, six (3.7%) of emotional or depression during the peripartum period, and two (1.2%) had a history of PTSD.

**Table 1:** Socio-demographic and obstetrical maternal characteristics for all patients and by EPDS status

	All patients		EPDS > 12 group		EPDS ≤12 group		*p*-value
	n^a^	n (% or mean ± sd); median [interquartile range]	n^a^	n (%) or mean ± sd; median [interquartile range]	n^a^	n (%) or mean ± sd; median [interquartile range]	
**Maternal socio-demographic characteristics**							
Age (years)	164	34.1 ± 4.8 34.0 [31.0; 37.0]	27	33.1 ± 4.8 33.0 [29.0; 37.0]	137	34.4 ± 4.8 34.0 [31.0; 37.0]	0.21^3^
Origin	154		24		130		0.11^2^
Caucasian		91 (59.1)		11 (45.8)		80 (61.5)	
Sub-Saharan		18 (11.7)		2 (8.3)		16 (12.3)	
North african		25 (16.2)		5 (20.8)		20 (15.4)	
Asian		6 (3.9)		2 (8.3)		4 (3.1)	
South American		3 (1.9)		2 (8.3)		1 (0.8)	
Other		11 (7.1)		2 (8.3)		9 (6.9)	
In a couple	156	148 (94.9)	24	24 (100)	132	124 (93.9)	0.61^2^
Educational Level	141		23		118		0.69^2^
Non graduate		16 (11.3)		3 (13)		13 (11)	
Precarity	161	6 (3.7)	26	1 (3.8)	135	5 (3.7)	1.00^2^
Socially isolated	160	5 (3.1)	26	1 (3.8)	134	4 (3.0)	1.00^2^
Isolated from family	30	8 (26.7)	1	1 (100)	29	7 (24.1)	0.27^2^
**Psychiatric history**							
Psychiatric or neurologic	163	6 (3.7)	27	1 (3.7)	136	5 (3.7)	1.00^2^
Postpartum depression	163	3 (1.8)	27	1 (3.7)	136	2 (1.5)	0.42^2^
Anxio-depressive syndrome or thymic pathology	162	6 (3.7)	27	1 (3.7)	135	5 (3.7)	1.00^2^
PTSD	163	2 (1.2)	27	0	136	2 (1.5)	1.00^2^
**Specific psychosocial contexts**							
Conflict within couple	162	2 (1.2)	27	0	135	2 (1.5)	1.00^2^
Transgenerational familial history of obstetrical pathologies	24	1 (4.2)	0		24	1 (4.2)	
Psychic vulnerability	162	15 (9.3)	27	3 (11.1)	135	12 (8.9)	0.72^2^
Psychosocial vulnerability	163	10 (6.1)	27	1 (3.7)	136	9 (6.6)	
**Obstetrical history**							
Pathological obstetrical history	161	10 (6.2)	26	1 (3.8)	135	9 (6.7)	1.00^2^
Perinatal death	163	3 (1.8)	27	0	136	3 (2.2)	1.00^2^
TOP	163	2 (1.2)	26	0	137	2 (1.5)	1.00^2^
Fetal malformation	163	1 (0.6)	27	0	136	1 (0.7)	1.00^2^
IUGR	164	6 (3.7)	27	1 (3.7)	137	5 (3.6)	1.00^2^
Maternal-fetal infection	162	0					
**Obstetrical characteristics**							
Primiparous	164	82 (50.0)	27	15 (55.6)	137	67 (48.9)	0.53^1^
Psychiatric or psychological follow-up	160	13 (8.1)	25	3 (12.0)	135	10 (7.4)	0.47^2^
Hypertension or pre-eclampsia	164	6 (3.7)	27	4 (14.8)	137	2 (1.5)	0.01^2^
Diabetes	164	22 (13.4)	27	0	137	22 (16.1)	0.03^2^
SGA fetus	162	8 (4.9)	26	2 (7.7)	136	6 (4.4)	0.62^2^
Premature delivery threat	164	5 (3.0)	27	3 (11.1)	137	2 (1.5)	0.03^2^
Maternal COVID-19 infection	164	3 (1.8)	27	2 (7.4)	137	1 (0.7)	0.07^2^
**Delivery**							
GA at delivery (WG)	164	39.3 ± 1.4 39.0 [39.0; 40.0]	27	39.0 ± 1.7 39.0 [38.0; 40.0]	137	39.3 ± 1.3 39.0 [39.0; 40.0]	0.45^3^
Partner present at delivery	117	67 (57.3)	19	9 (47.4)	98	58 (59.2)	0.34^1^
Mode of delivery	164		27		137		0.08^2^
Spontaneous vaginal		89 (54.3)		9 (33.3)		80 (58.4)	
Assisted vaginal		32 (19.5)		7 (25.9)		25 (18.2)	
Emergency c-section		25 (15.2)		7 (25.9)		18 (13.1)	
Elective c-section		18 (11.0)		4 (14.8)		14 (10.2)	
Episiotomy	164	12 (7.3)	27	2 (7.4)	137	10 (7.3)	1.00
Perineal tear	164	87 (53.0)	27	10 (37.0)	137	77 (56.2)	0.07^1^
Postpartum hemorrhage	164	15 (9.1)	27	3 (11.1)	137	12 (8.8)	0.72^2^
**Neonate**							
Neonatal complications	164	25 (15.2)	27	9 (33.3)	137	16 (11.7)	0.008
Neonatal COVID-19 infection	162	1 (0.6)	27	1 (3.7)	135	0	0.17^2^

*EPDS* Edinburgh Postnatal Depression Scale

^a^ available data

*sd* Standard deviation, *PTSD* Post-traumatic stress disorder, *TOP* Termination of pregnancy, *IUGR* Intra uterine growth retardation, *SGA* Small for gestational age, *GA* Gestational age WG: weeks of gestation

### Obstetrical data and perinatal outcome

Half of the patients were primiparous (Table 1). Maternal hypertension/preeclampsia or diabetes were observed in six (3.7%) and 22 (13.4%) of the women, respectively. Five (3%) of the women had threatened preterm labor. COVID-19 infection occurred during the pregnancy in three women (uncomplicated maternal infection with no maternal hospitalization). Among these, one neonate had a positive COVID-19 polymerase chain reaction test (no neonatal signs of infection). The mean gestational age at delivery was 39 weeks (range: 34–42 weeks). Spontaneous labor occurred in 101 of the cases (69.7%) and the overall vaginal delivery rate was 54.3% (Table 1). The partner was present at the delivery in 67/117 cases (57.3%). Of the 25 newborns (15.2%) who presented with neonatal complications, 18 had neonatal hyperbilirubinemia. Neonatal hospitalization was required for 10 newborns (hyperbilirubinemia, extreme prematurity, feeding disorders).

### Maternal psychological outcome

Major PPD (EPDS score > 12) was observed in 27/164 women 16.5% [95%CI: 11.14–23.04] either at Day 10 or 6–8 weeks postpartum. This was similar to the rate of 15% reported prior to the pandemic (*p* = 0.6). Mild to major depression (EPDS > 10 at one of the two telephone interviews) was observed in 40/164 women (24.4%) [95%CI:18.03–31.70].

Maternal hypertension/preeclampsia (*p* = 0.007), emergency cesarean section (*p* = 0.03) and neonatal complications (*p* = 0.008) were significantly associated with an EPDS > 12 in both univariate and multivariate analysis (OR = 10 [95%CI: 1.5–68.7], OR = 4.09 [95%CI: 1.2–14.1], OR = 4.02 [95%CI: 1.4–11.6], respectively) (Table 2). Threatened preterm labor was significantly associated with PPD (p = 0.03) and was an associated factor with maternal hypertension/preeclampsia.

**Table 2:** Multivariate analysis of risk factors for postpartum depression (EPDS > 12)

	OR [95% CI]	*P*-value	AOR [95% CI]	*P*-value
Hypertension or pre-eclampsia	11.74 [2.03 — 67.82]	0.01	10.01 [1.46 — 68.67]	0.02
Threatened preterm labor	8.44 [1.34 — 53.19]	0.02		
Maternal COVID-19 infection	10.88 [0.95 — 124.6]	0.06		
Delivery				
Spontaneous vaginal	Reference		Reference	
Assisted vaginal	2.49 [0.84 — 7.37]	0.10	2.29 [0.73 — 7.22]	0.16
Emergency c-section	3.46 [1.14 — 10.51]	0.03	4.09 [1.18 **—** 14.13]	0.03
Elective c-section	2.54 [0.69 — 9.39]	0.16	2.48 [0.61 — 10.08]	0.20
Neonatal complications	3.78 [1.46 — 9.83]	0.01	4.02 [1.40 **—** 11.55]	0.02

*EPDS* Edinburgh Postnatal Depression Scale, *OR [95% CI]* Odds ratio [95% Confidence Interval], *AOR* Adjusted Odds ratio

The median PPQ score was 3.5 ± 2.5 and 17 women (12%) had a PPQ score above 6. Thirty-four women (20.7% [95%CI: 14.8–0.28]) had post traumatic-depressive symptoms with a combination of major depression (EPDS score > 12) and/or a high risk of PTSD (PPQ > 6) at either day 10 or 6–8 weeks postpartum.

Mean scores of satisfaction in the couple were high and stable between the two interviews: the DAS-16 total score was 62.7 (± 8.4) at Day 10, and 62.6 (± 9.1) at 6–8 weeks postpartum. The perception of a good relationship in the couple (DAS total score, and DA and QI sub-score) was negatively correlated with the EPDS scores (DAS total x EPDS, *ρ* = − 0.22, *p* = 0.01; DA x EPDS; *ρ* = − 0.25389, *p* = 0.005; QI x EPDS: ρ = − 0.15329 *p* = 0.0932). Some maternal-newborn interactions were slightly impaired with a mean MIBS score of 1.6 (± 1.7) at Day 10 and 1.3 (± 1.4) at 6–8 weeks. In the French MIBS validation study, an absence of interaction disorder represented a score of 0 to 1 (20). The MIBS scores were correlated with the global EPDS and PPQ score at Day 10 (*ρ* = 0.32, *p* = 0.0003, and *ρ* = 0.20, *p* = 0.02, respectively). PPD (EPDS > 12 at Day 10) and post-partum traumatic symptoms (PPQ scores) were also strongly correlated (*ρ* = 0.6, *p* < 0.0001).

### Factors associated with an increased risk of maternal depression and post-traumatic stress syndrome

Thirty-four (20.7%) patients had an EPDS > 12 or PPQ > 6. The variables significantly associated with depressive-anxiety symptoms were: the geographic origin of the mother (*p* = 0.04), maternal hypertension/preeclampsia (*p* = 0.02), partner not present at delivery (*p* = 0.05), neonatal complications (*p* = 0.04), and maternal COVID-19 infection during the pregnancy (*p* = 0.008). Adjusted multivariate analysis found that the variables that were significantly associated with post traumatic-depressive symptoms were: a previous child hospitalization (OR = 7.49 [95%CI: 1.61–34.94] *p* = 0.010), neonatal complications (OR = 3 [95%CI: 1.08–8.35] *p* = 0.04), hypertension/preeclampsia (OR = 8.17 [95%CI: 1.245–3.70] *p* = 0.03) and emergency cesarean section (OR = 2.08 [95%CI: 1.10–10.85] *p* = 0.05) (Table 3).

**Table 3:** Multivariate analysis of risk factors associated with EPDS > 12 and/or PPQ > 6

	OR [95% CI]	*P*-value	AOR [95% CI]	*P*-value
Previous child hospitalization	4.13 [0.98 — 17.48]	0.05	7.49 [1.61 — 34.94]	0.01
Hypertension or pre-eclampsia	8.40 [1.47 — 48.02]	0.02	8.17 [1.24 — 53.70]	0.03
Threatened preterm labor	6.09 [0.98 — 38.05]	0.05		
Mode of Delivery				
Spontaneous vaginal	Reference		Reference	
Assisted vaginal	1.90 [0.70 — 5.13]	0.06	2.19 [0.75 — 6.39]	0.15
Emergency c-section	2.68 [0.96 — 7.48]	0.03	3.45 [1.10 — 10.85]	0.03
Elective c-section	2.19 [0.67 — 7.18]	0.20	2.34 [0.65 — 8.50]	0.20
Neonatal complications	2.52[1.00 — 6.35]	0.05	3.00 [1.08 — 8.35]	0.04

*EPDS* Edinburgh Postnatal Depression Scale, *PPQ* Post-traumatic Stress Disorder Questionnaire, *OR [95% CI]* Odds ratio [95% Confidence Interval], *AOR* Adjusted Odds ratio

### Vulnerability screening and psychological monitoring

Psychological support was offered to 71/164 women (42%), including 46 patients (28%) with an EPDS score > 10 and/or PPQ > 6. In the other 25 cases, the scores did not reach the risk thresholds, but the free speech interviews revealed clinical indicators of vulnerability. Psychological or psychiatric interventions were organized for 21 of the 71 women. In the other 50 patients, a follow-up was proposed based on regular phone calls by psychologists. Among these, an improvement was observed in 20 cases with no sign of depression at 6–8 weeks. For the remaining 30 patients, 18 opted for outpatient psychological support and 12 were lost to follow-up.

## Discussion

### Main findings

The rate of 16.5% of women with major PPD (EPDS score > 12) we found either at Day 10 or 6–8 weeks postpartum was similar to the one observed in our population prior to the COVID-19 pandemic [8]. The risk factors associated with major PPD in our population were not specifically associated with the COVID-19 pandemic. Similarly, couple’s adjustment and mother child-interaction showed overall a rather good functioning. However, depressive and/or post-traumatic symptoms were observed in 20.8% of the cases and in the event of emotional post-partum impact, the mother-child interaction, as captured by the MIBS, and the couple’s adjustment, as captured by the DAS, were also impaired. To support women during this period of increased vulnerability, we offered an adapted psychological follow-up to 71/164 (43%) of the mothers. This support led to clinical improvement in 20 cases.

### Strengths and limitations

One of the strengths of our study lies in its prospective design. Furthermore, we did not rely solely on self-questionnaires during the calls but included a time of free exchange with a psychologist to increase the chances of picking up on signs of psychological vulnerability. In addition, we screened patients at risk of PPD at two separate times up to 6–8 weeks postpartum. Finally, psychological support was offered to improve the immediate psychological outcome of the patients. One of the weaknesses of the study is related to the fact that we did not obtain the calculated sample size for the study. This limitation was partly due to a delay in obtaining regulatory approval, which reduced our inclusion period to 6 weeks. In addition, we were unable to capture the reason for which some patients who gave birth during this period declined to participate in the study. Another limitation is the 16% attrition rate we observed between the two separate times of evaluation. In addition, we were unable to complete the follow-up for all the 71 women in whom psychological vulnerability was identified at Day 10. Finally, we did not include a pre-lockdown control group. Instead, we compared the rate of depression with the one in the largest multisite Parisian epidemiological study [8]. We believe this rate plausible as a previous study from our group, assessing how pre/perinatal depression (as opposed to post-natal depression) affected infants’ development, reported the same rate (20%) of depression based on the EPDS [21].

### Comments

Contrary to several studies carried out throughout the world, we did not observe a significant increase in the PPD rate during the period of the first strict lockdown of 2020. Our PPD rate of 16% apparently contradicts several recent reports supporting an increased risk of perinatal depression related to the COVID-19 pandemic with rates ranging between 21 and 56.9% [2, 22–25]. Our rate is closer to the rate of 12% found in the Netherlands, even if this rate was twice as high as their pre-pandemic rate [26], and higher than the 6.4% reported in Silverman et al.’s study in New York City (Mount Sinai Health System Sites), USA [27]. It should be noted that most of the studies, often performed at different times during the postpartum period, relied on self-completed online questionnaires, which may introduce a bias. Furthermore, it should be noted that the EPDS questionnaire is merely a screening method, which does not replace a clinical diagnosis of depression [28]. It is also difficult to compare studies because of the heterogenous characteristics of the studied populations, including the socio-economic level. However, Chmielewska et al. explored this large heterogeneity between studies in their meta-analysis [1]. The subgroup analyses according to a country’s income status showed a statistically significant increase in mean EPDS scores in low- and middle-income countries but not in high-income countries. We found that the rate of anxiety-depressive disorders associated with post-traumatic stress was 20.7%. This is in line with the increase in anxiety reported both during pregnancy and in the postpartum period in the two previously cited meta-analyses [1, 5].

Variables associated with increased PPD from univariate analysis include well-known risk factors which were unrelated to the pandemic such as sociodemographics (low income, migration [12, 29]), somatic conditions (hypertension/preeclampsia [30, 31]), and childbirth complications [32, 33]. Although hypertension was associated with an excess risk of complications in case of COVID infection early on in the pandemic, it is impossible to link the hypertension factor with the crisis. Similarly, the impact of the partner’s absence at the delivery was not associated in our study with an increased risk of PPD. However, it did appear to be associated with an increased risk of anxiety-depressive disorders. Finally, an emergency cesarean section was associated with an increased risk of PPD and post-traumatic syndrome. Even if this factor was already known, it could be considered to be related to the pandemic since partners were not allowed in the operating rooms during the period of the first lockdown in our maternity unit, as in most units in France. Recently, Berthold et al. reported an original study assessing the impact of the COVID-19 lockdown on perinatal experience measured with the Labour Agency Scale. As they included a pandemic control group without restriction, they were able to disentangle some questions raised above. The perinatal experience was indeed negatively affected in women exposed to lockdown restrictions or restrictions during childbirth, but also in women financially impacted by the pandemic and in women who had a caesarian delivery [34].

Regarding family correlates, MIBS and DAS results confirm the links between maternal emotional disorders, couple relationship and parent-child interactions as emotional post-partum dysfunction were significantly correlated with both poor mother-child interaction and poor couple’s adjustment [10, 35]. Therefore, treating peripartum depression has benefits for women themselves but also prevents eventual consequences in children [21]. In addition, attention towards the couple’s relationship, specifically the degree of accordance between future mothers and fathers, appears very relevant to look for high risk pregnant women [18, 19]. This also means more attention towards fathers [35].

We are aware that our study was not designed to assess the impact of our prevention measures. However, the fact that we did not observe an increase in the PPD rate in this period could also be related to the call program we set up during the first lockdown period. By calling the mothers at Day 10 and then at 6–8 weeks, we were able to identify mental health vulnerability in 71/164 patients. We consequently organized conventional psychological care programs for 21 patients and regular phone calls by psychologists with the remaining 50, which resulted in an improvement in the psychological state for at least 20 mothers.

## Conclusions

In our population we did not observe an increased rate of major PPD during the first COVID-19 lockdown period. However, anxiety-depressive signs were identified in up to 20% of our patients. The organization we set up consisting of an evaluation by psychologists at two time points during the postpartum period enabled us to support the mothers thereby limiting the psychological consequences of the social restriction measures in place.

## Data Availability

The data used and analyzed during the current study are available from the promotor, APHP.

## Abbreviations

PPD: Postpartum depression
EPDS: Edinburgh Posnatal Depression Scale
PPQ: Perinatal Post-traumatic Stress Disorder Questionnaire
CI: Confidence Interval
OR: Odds ratio
IUGR: Intra-uterine growth restriction
PTSD: Post-traumatic stress disorder
DAS: Dyadic Adjustment Scale
DA: Degree of accordance
QI: Quality if interaction
MIBS: Mother-Infant Bonding Scale
USA: United States of America
